# Targeting the radiation-induced ARv7-mediated circNHS/miR-512-5p/XRCC5 signaling with Quercetin increases prostate cancer radiosensitivity

**DOI:** 10.1186/s13046-022-02287-4

**Published:** 2022-08-03

**Authors:** Dong Chen, Fu-Ju Chou, Yuhchyau Chen, Chi-Ping Huang, Hao Tian, Yaqin Wang, Yuanjie Niu, Bosen You, Shuyuan Yeh, Nianzeng Xing, Chawnshang Chang

**Affiliations:** 1 Department of Urology and State Key Laboratory of Molecular Oncology, National Cancer Center/National Clinical Research Center for Cancer/Cancer Hospital, Chinese Academy of Medical Sciences and Peking Union Medical College, 100021 Beijing, China; 2 George Whipple Lab for Cancer Research, Departments of Pathology, Urology, Radiation Oncology and The Wilmot Cancer Institute, University of Rochester Medical Center, Rochester, NY 14642 USA; 3 Department of Urology, China Medical University, Taichung, 404 Taiwan; 4 Tianjin Institute of Urology, Tianjin Medical University, Tianjin, 300211 China; 5 Key Laboratory of Cardiovascular Epidemiology Department of Epidemiology National Center for Cardiovascular Diseases, Fuwai Hospital, Chinese Academy of Medical Sciences and Peking Union Medical College, 100037 Beijing, China; 6 Department of Urology, Shanxi Province Cancer Hospital/Shanxi Hospital Affiliated to Cancer Hospital, Chinese Academy of Medical Sciences/Cancer Hospital Affiliated to Shanxi Medical University, Taiyuan, 030013 China

**Keywords:** ARv7, circNHS, miR-512-5p, XRCC5, Quercetin, Prostate cancer, Radiosensitivity

## Abstract

**Background:**

Radiation therapy (RT) with androgen deprivation therapy (ADT) is an effective therapy to suppress the locally advanced prostate cancer (PCa). However, we unexpectedly found that RT could also induce the androgen receptor splice variant 7 (ARv7) expression to decrease the radiosensitivity**.**

**Methods:**

The study was designed to target ARv7 expression with Quercetin or ARv7-shRNA that leads to enhancing and increasing the radiation sensitivity to better suppress the PCa that involved the modulation of the circNHS/miR-512-5p/XRCC5 signaling.

**Results:**

Mechanism studies revealed that RT-induced ARv7 may function via altering the circNHS/miR-512-5p/XRCC5 signaling to decrease the radiosensitivity***. ***Results from preclinical studies using multiple in vitro cell lines and in vivo mouse models concluded that combining RT with the small molecule of Quercetin to target full-length AR and ARv7 could lead to better efficacy to suppress PCa progression.

**Conclusion:**

Together, these results suggest that ARv7 may play key roles to alter the PCa radiosensitivity, and targeting this newly identified ARv7 mediated circNHS/miR-512-5p/XRCC5 signaling with Quercetin may help physicians to develop a novel RT to better suppress the progression of PCa.

**Supplementary Information:**

The online version contains supplementary material available at 10.1186/s13046-022-02287-4.

## Background

Prostate cancer (PCa) is the second leading cause of cancer deaths and the most common cancer in men in the United States [1] and its morbidity is also rapidly rising in Asian countries [2]. Radiation therapy (RT) including external beam radiotherapy (EBRT) and brachytherapy, is one of the standard treatment modalities for managing primary or locally advanced disease [3]. However, there are still 20–25% of PCa patients with noninvasive disease (stage T1-T2) relapse after high doses of radiation in less than 5 years [1]. Clinically, the combination of RT and androgen deprivation therapy (ADT) is superior to RT alone for treatment of patients with localized, intermediate-risk and high-risk PCa, since ADT may abrogate the androgens/androgen receptor (AR)-induced DNA damage response (DDR) system so that RT can be more efficient to suppress PCa [4, 5].

Despite use of combined ADT and RT, biochemical recurrence was up to 50% in high metastatic risk PCa patients [6]. One possible mechanism proposed to explain this was that ADT treatment could induce the expression of AR variants (ARVs), especially the AR variant 7 (ARv7) in PCa [7–9] and ARVs could mediate DDR following RT [10]. Therefore, it is necessary to inhibit both full-length AR and ARv7 signal pathway to effectively improve PCa radiosensitivity. Moreover, there are limited reports on the effect of ionizing radiation (IR) on ARVs, especially ARv7 expression in PCa.

Quercetin (Que, 3,3',4',5,7-pentahydroxyflavone) is a bioactive plant-derived flavonoid, abundant in fruits and vegetables, that has been used as a nutritional supplement in several countries [11]. Daily human intake of Que ranges from 10 to 100 mg depending on different dietary habits, and it can reach 500–1,000 mg if selected highly purified extracts are used [12]. Recent studies also indicated that Que might have anti-cancer properties that could suppress cell growth in many types of cancer cell lines and in vivo models [13–15]. Its linkage to the ARVs, especially to the IR-induced ARv7 during RT, however, remains unclear.

Circular RNAs (circRNAs) are highly conserved and stable covalently closed RNA transcripts generated by back-splicing of a single pre-mRNA with gene-regulatory potential [16, 17]. Emerging evidence shows that circRNAs are closely related to human diseases, especially cancers, and may act as better biomarkers due to their abundance and stability [18, 19]. The circRNAs have also been linked to the radiosensitivity of several cancer types [20, 21] and we previously found circZEB1 could decrease radiosensitivity by mediating the miR-141-3p/ZEB1 signaling pathway in PCa cells [22].

Here we found that IR may function via inducing the ARv7 expression to decrease the subsequent RT efficacy, and adding Que may then suppress both IR-induced full-length AR and ARv7 to better increase the PCa radiosensitivity.

## Methods

### Cell lines and cell culture

C4-2, CWR22Rv1(22Rv1), VCaP and the 293T cell lines were obtained from American Type Culture Collection. C4-2, 22Rv1 and VCaP cells were maintained in RPMI 1640 medium and 293T cells in DMEM media, all containing 10% fetal bovine serum (FBS), antibiotics (100 units/ml penicillin and 100 µg/ml streptomycin), and 2 mM glutamine (Invitrogen) in a humidified 5% CO2 environment at 37 °C. All cell lines were characterized and authenticated as bacteria and mycoplasma free following ATCC’s instructions.

### Plasmids and lentivirus

The pLKO.1-shAR, pLKO.1-shARv7, pLKO.1-circNHS, pLKO.1-XRCC5, pWPI-ARv7, pWPI-circNHS and pWPI-XRCC5 plasmids, the psPAX2 packaging plasmid, and pMD2G envelope plasmid (lentivirus:packaging:envelope, 2:1:1) were cotransfected into 293 T cells using the standard calcium chloride transfection method for 48 h to obtain the lentiviral supernatant. For the virus infection, supernatants were added to the target cells with polybrene to prepare stable cell line clones.

### Clonogenic survival assays

Transfected cells were irradiated with increasing doses (2–8 Gy) delivered as a single dose using the Cs137 γ-irradiator. Clonogenic survival assays were carried out as previously described [22].

### Quantitative real-time PCR

Total RNA was isolated using TRIzol® Reagent (#15596026 ThermoFisher) and was reverse transcribed into cDNA using the iScript™ cDNA Synthesis Kit (# 1,708,891, Bio-Rad). The primer sequences are listed in Table S1. The qRT-PCR was performed using a Bio-Rad iQ5 real-time thermal cycler and iQ™ SYBR® Green Supermix (#1708880, Bio-Rad). Relative mRNA expression levels were normalized against GAPDH levels (as an internal control).

### Western blot assays

For western blot analyses, protein extracts of each sample (50 µg/lane) were electrophoretically separated and transferred onto PVDF membranes. After blocking membranes with 5% non-fat milk TBST solution, they were incubated with appropriate dilutions of specific primary antibodies against ARv7 (#ab198394, abcam, USA), AR (#sc-816, Santa Cruz, USA), γ-H2AX (#05–636 clone JBW301, EMD Millipore), XRCC5 (#sc-5309, Santa Cruz, USA), SPRTN (#PA5-110,424,Thermofisher, USA) and GAPDH (#sc-166, Santa Cruz, USA), followed by horseradish peroxidase-conjugated secondary antibody. The blots were then incubated with HRP-conjugated secondary antibodies and were detected by SuperSignal™ West Femto Maximum Sensitivity Substrate (#34,095, ThermoFisher Scientific) using the Bio-Rad imaging system.

### Neutral comet assay

Double strand breaks (DSBs) were assessed by single-cell gel electrophoretic comet assay kits (Trevigen) under neutral conditions according to the manufacturer’s protocol. Briefly, cells were harvested at the indicated times after a single dose of 6 Gy irradiation, mixed with 0.7% low melting point agarose, and plated on CometSlide microscope slides, followed by cell lysis step. Cells then subjected to electrophoresis under neutral conditions and were stained with SYBR Gold (S11494, Thermo Fisher Scientific). Comets were visualized using a Zeiss fluorescent microscope and were quantified followed by analysis using CASP Comet Assay Software.

### Chromatin immunoprecipitation assay

Cells were cross-linked with 4% formaldehyde for 10 mins followed by cell collection and sonication with a predetermined power to yield genomic DNA fragments 300–1000 bp in length. Lysates were precleared sequentially with normal rabbit IgG (#sc-2027, Santa Cruz Biotechnology) and Protein A/G. Anti-ARv7 antibody (2.0 µg) was added to the cell lysates and incubated at 4 °C overnight. As a negative control, IgG was used in the reaction. PCR products were analyzed by agarose gel electrophoresis.

### Luciferase reporter assay

The human promoter region of the NHS 5’ promoter was constructed into the pGL3-basic vector (Promega). Site-directed mutagenesis of the ARv7 binding site in the NHS 5' promoter was achieved with the Quick-Change mutagenesis kit. C4-2 cells prepared as ionizing radiation resistant (IRR) cells (C4-2–IRR cells were plated in 24-well plates, and the cDNAs were transfected with Lipofectamine 3000 transfection reagent (Invitrogen) according to the manufacturer’s instructions. Luciferase activity was measured 48 h after transfection by the Dual-Luciferase Reporter Assay System (Promega) according to the manufacturer’s manual.

### Pull-down assay with biotinylated circNHS probe

In brief, 1 × 10^7^ C4-2-IRR cells were harvested, lysed, and sonicated. The cell lysate mixture was rotated overnight at 4 °C after adding 2 µL RNase inhibitor and 500 pmol/L biotin-labeled antisense oligo against circNHS. The lysate mixture was rotated for 2 h at 4 °C after adding 10 µL streptavidin agarose beads (No. 88817, Pierce Biotechnology). Total RNA was extracted by TRIzol (Invitrogen) according to the manufacturer's protocol, reverse transcribed, and subjected to qPCR analysis to detect the miRNAs.

### RNA in situ hybridization

The biotin-labeled circNHS probe was designed and synthesized by IDT (Integrated DNA Technologies, USA). The probe signal was detected by Alexa Fluor™ 594 Tyramide SuperBoost™ Kit (Thermo Fisher Scientific, USA) according to the manufacturer’s instructions.

### RNA isolation

The subcellular localization of circNHS was detected using the PARIS™ Kit according to the manufacturer's protocol (Thermo Fisher Scientific, USA).

### Actinomycin D assay

C4-2-IRR and 22Rv1 cells were seeded at 1 × 10^5^ cells per well in a 6-well plate overnight and then exposed to 2 mg/L actinomycin D (Sigma, USA). The cells were harvested and the stability of circNHS was analyzed using qRT-PCR.

### Xenograft mouse models

To test the effects of the combination of Que and IR in tumor growth in vivo, we used the ARv7 positive 22Rv1 cell line to generate human xenograft tumors in nude mice. 22Rv1 cells (1 × 10^6^) were injected subcutaneously into the left flanks of 6-week-old nude mice (National Cancer Institute). When xenografted tumors reached approximately 150 mm^3^, mice were randomized into four groups (six mice/group): (a) vehicle alone (NC group), (b) 8 Gy radiation alone (IR group), (c) 75 mg/kg Que alone (Que group), and (d) 75 mg/kg Que combined with irradiation (IR + Que). The groups (b) and (d) animals were given a single dose of Que by i.p. injection 6 h before irradiation. For the irradiation of tumors (group b and d), mice were anesthetized i.p. with Avertin (when irradiated) or isoflurane (when measuring tumor size). Xenografts were locally irradiated with a Cs137 irradiator (URMC, Rochester, NY), while other body parts were protected with lead shielding. Tumor sizes were measured weekly with calipers, and tumor volumes were estimated using the following formula: volume = (length x width x width)/2. All animal experiments were performed in accordance with the guidelines and with the approval of the University of Rochester Department of Laboratory Animal Medicine.

### Statistical analysis

Unless otherwise stated, all data are shown as the mean ± standard error of the mean (SEM). Statistical analyses were performed using SPSS 23.0 statistical software or GraphPad Prism 7 (GraphPad Software, Inc., La Jolla, CA). Comparisons between multiple groups were calculated by repeated measures analysis of variance (ANOVA) with Bonferroni corrected t-tests, and comparisons between two groups were calculated by Student t-test to determine significance. A P-value of < 0.05 was considered statistically significant.

## Results

### IR-induced ARv7 expression led to decrease radiosensitivity

**Fig. 1:**
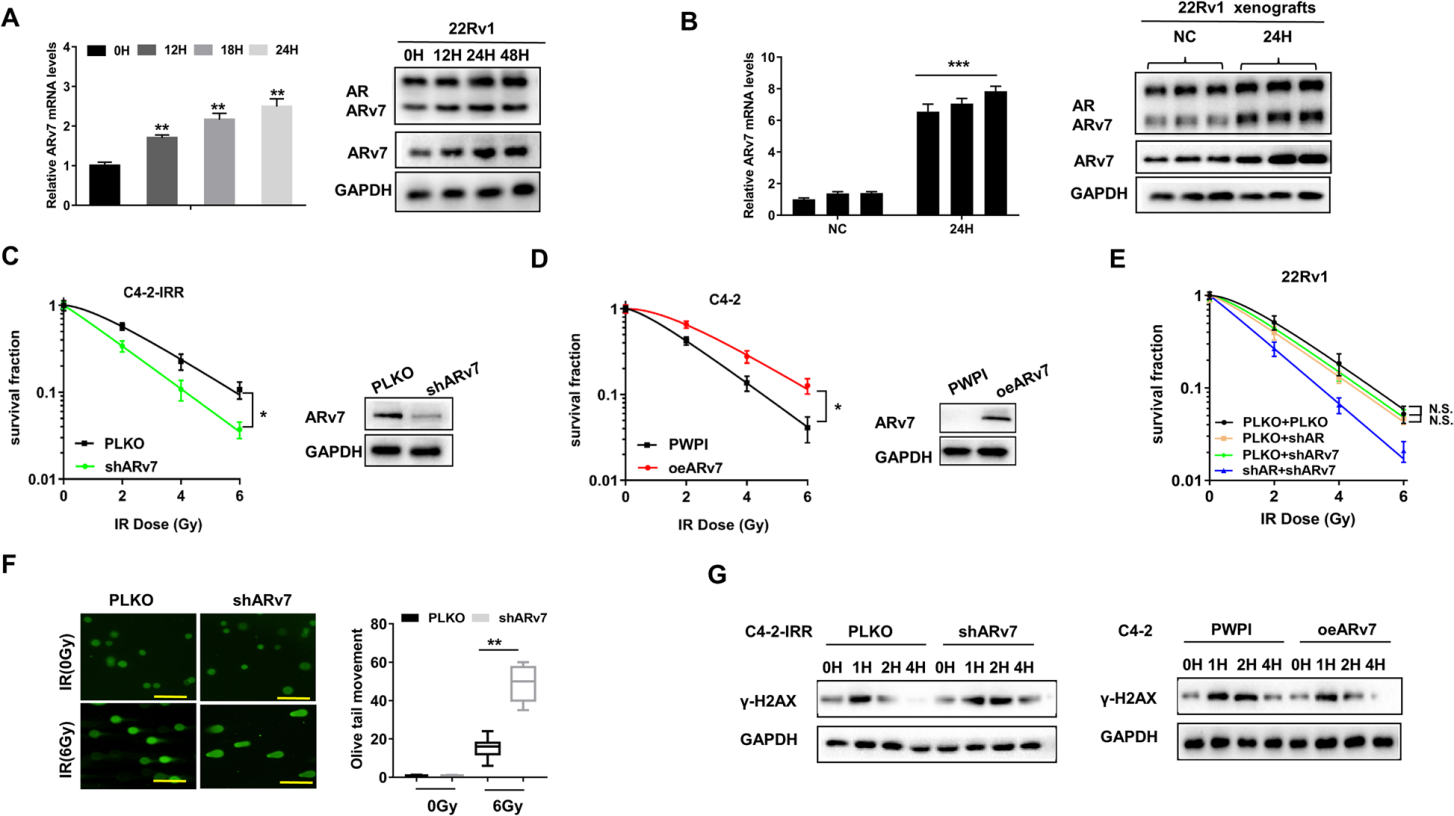
**A** ARv7 mRNA and protein levels in 22Rv1 cells after 4 Gy IR treatment. **B** ARv7 mRNA and protein levels in 22Rv1 xenografts after 4 Gy IR treatment. **C** C4-2-IRR parental cells and C4-2-IRR-shARv7 cells were cultured with 5 µM Enz for 24 h, then treated with 0-6Gy IR. **D **The oeARv7 effects on C4-2 cells survival after 0-6 Gy IR using clonogenic assay. **E** The shARv7 and/or shAR effects on 22Rv1 cells survival after IR using clonogenic assay. **F** shARv7 effects on DNA damage in C4-2-IRR cells after IR using comet assay. Scale bar = 10 µm. **G** WB analysis of γ-H2AX in different groups. The oeARv7 can decrease DNA damage repair time in C4-2 cells and shARv7 can increase DNA damage repair time in C4-2-IRR cells. Data are presented as mean ± SEM. *P < 0.05, **P < 0.01, ***P < 0.001, compared with the controls. N.S., not significant

Recent studies indicated that ADT treatment would induce the expression of ARv7 [23, 24], however, the potential impact of IR treatment on ARv7 expression, remains unclear. Here we first developed the radioresistant C4-2-IRR cells via treating parental C4-2 cells with repeated 2 Gy radiation. Radioresistance of cells was confirmed in clonogenic assays by showing higher survival of C4-2-IRR cells than respective parental cells at indicated radiation dose and clonogenic survival of C4-2-IRR cells were not affected by 5μM enzalutamide (Enz) (Fig. S1A). Interestingly, ARv7 was significantly upregulated in C4-2-IRR cell lines than in the parental cells (Fig. S1B). Moreover, we found that IR could significantly increase the ARv7 expression at the protein and mRNA levels in ARv7-positive 22Rv1 cells and xenografts (Fig. 1A and B).

To further examine if IR may function via inducing the ARv7 to increase radioresistance, Enz was added to a final concentration of 5μM to inhibit AR activity, then we knocked down ARv7 with shARv7 in C4-2-IRR cells or ectopic overexpression of ARv7 in C4-2 cells. The results revealed that suppressed ARv7 in C4-2-IRR cells led to increase the PCa radiosensitivity (Fig. 1C). In contrast, increased ARv7 in C4-2 cells could then increase the radioresistance of PCa cells (Fig. 1D). As 22Rv1 cells have both ARfl and ARv7, we then applied the shAR and/or shARv7 to suppress the expression of ARfl and/or ARv7. The results revealed such suppression of ARfl alone or ARv7 alone could not decrease clonogenic survival following IR (Fig. 1E), and only knock down of both ARfl and ARv7 led to decrease the clonogenic survival following IR (Fig. 1E). Together, these results suggest that suppression of both ARfl and ARv7 could lead to better effect to improve PCa radiosensitivity.

To further strengthen above conclusion, we added 5uM Enz to inhibit AR activity, then knocked down ARv7 with shARv7 in C4-2-IRR cells or ectopically overexpressed  ARv7 in C4-2 cells. Next, we examined the potential impact of ARv7 on PCa cells DNA repair by comet assays and the results from these assays also revealed that decreasing ARv7 in C4-2-IRR cells significantly increased DNA fragmentation following IR (Fig. 1F). Similar results from WB (Fig. 1G, left) analysis of the γ-H2AX levels showed that decreasing ARv7 in C4-2-IRR cells increased the DNA damage repair time. In contrast, increasing ARv7 resulted in less time to complete DNA damage repair in the C4-2 cells (Fig. 1G, right).

Together, results from Fig.1 suggest that IR-induced ARv7 may decrease the continued RT efficacy.

### Mechanism dissection of how ARv7 can alter the radiosensitivity*:* via increasing circNHS expression.

**Fig. 2:**
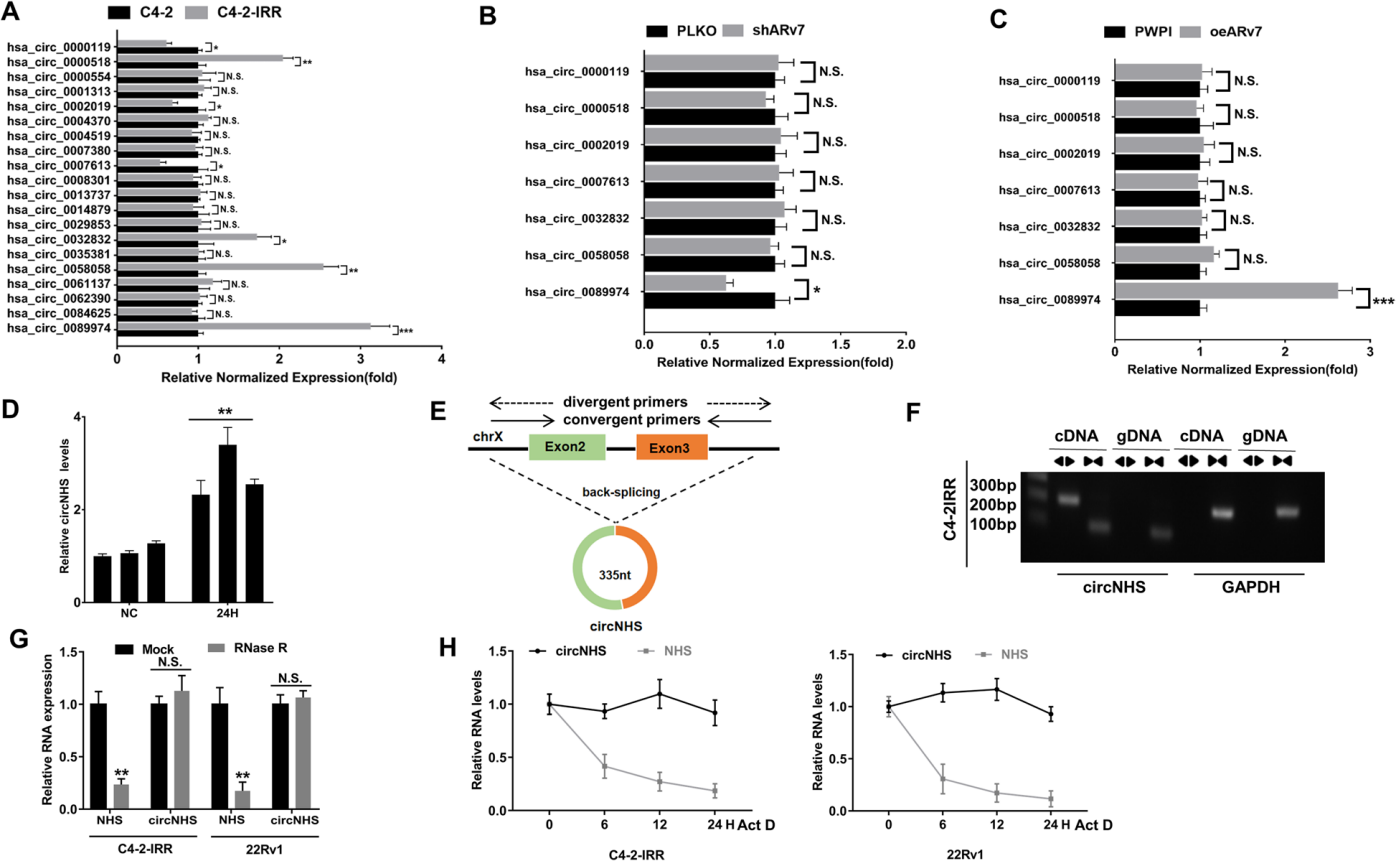
**A** PCR analysis of 20 circRNAs levels in C4-2 parental cells and C4-2-IRR cells. **B** Knocking down ARv7 with shARv7 in C4-2-IRR cells and PCR analysis of 7 circRNAs levels. **C** oeARv7 in C4-2 parental cells and PCR analysis of 7 circRNAs levels. **D** circNHS levels in 22Rv1 cells xenografts after 4 Gy IR treatment. **E** Schematic diagram of the genomic location and splicing pattern of circNHS. **F** The existence of circNHS was validated in C4-2 parental cells and 22Rv1 cells by PCR. Divergent primers amplified circNHS from cDNA, but not from genomic DNA (gDNA). GAPDH was used as a negative control. **G** PCR analysis of RNase R treatment assay to confirm the circNHS formation is a circRNA sequence. **H** The relative RNA levels of circNHS and NHS were analyzed by RT-qPCR after treatment with Actinomycin D at the indicated time points. Data are presented as mean ± SEM. *P < 0.05, **P < 0.01, ***P < 0.001 compared with the controls. N.S., not significant

To further dissect the mechanism of how ARv7 can alter the radiosensitivity, we focused on circRNAs, as recent studies indicated that the expression of selected circRNAs could be altered after IR exposure [20, 21]. From circRNA Array data in radioresistant cancer cells [25], we first chose the 10 most upregulated/downregulated circRNAs and detected their expression in C4-2-IRR cells model. The RT-qPCR results revealed that 7 of those 20 circRNAs had significant changes in C4-2-IRR Cells compared with C4-2 parental cells (Fig.  2A). Knocking down ARv7 (Fig. 2B) and ectopically overexpressing ARv7 (Fig. 2C) led to significantly regulated the expression of circNHS (hsa_circ_0089974) in PCa cells. Moreover, we found that IR could significantly increase circNHS levels in 22Rv1 xenografted tumors (Fig. 2D).

Next, results from circBase database analysis indicated that circNHS is generated from back-splicing of two exons (exon 2 and exon 3) of the NHS gene (chrX:17,705,861–17,710,588) (Fig. 2E). To avoid trans-splicing or genomic rearrangements, including head-to-tail splicing, we then applied multiple approaches to rule out such possibilities. We first designed convergent primers to amplify NHS mRNA and divergent primers to amplify circNHS. Using cDNA and genomic DNA from C4-2-IRR and 22Rv1 cell lines as templates, circNHS was amplified from cDNA only by the divergent primers, whereas no amplification product was observed from genomic DNA (Fig. 2F). Furthermore, the results from the RNase R assay revealed that circNHS is the circular form, with better resistance to RNase R digestion than linear NHS (Fig. 2G). Finally, the results from adding actinomycin D to inhibit transcription also indicated that the half-life of circNHS is longer and more stable than NHS mRNA in C4-2-IRR and 22Rv1 cells (Fig. 2H). Finally, the circRNA microarrays data from Yang et al. [26] showed that circNHS was highly expressed in high-grade (Gleason > 8) PCa tissues when compared with low-grade (Gleason < 6) PCa tissues (Fig. S1C).

### ARv7 increases circNHS expression via altering the transcriptional regulation

**Fig. 3:**
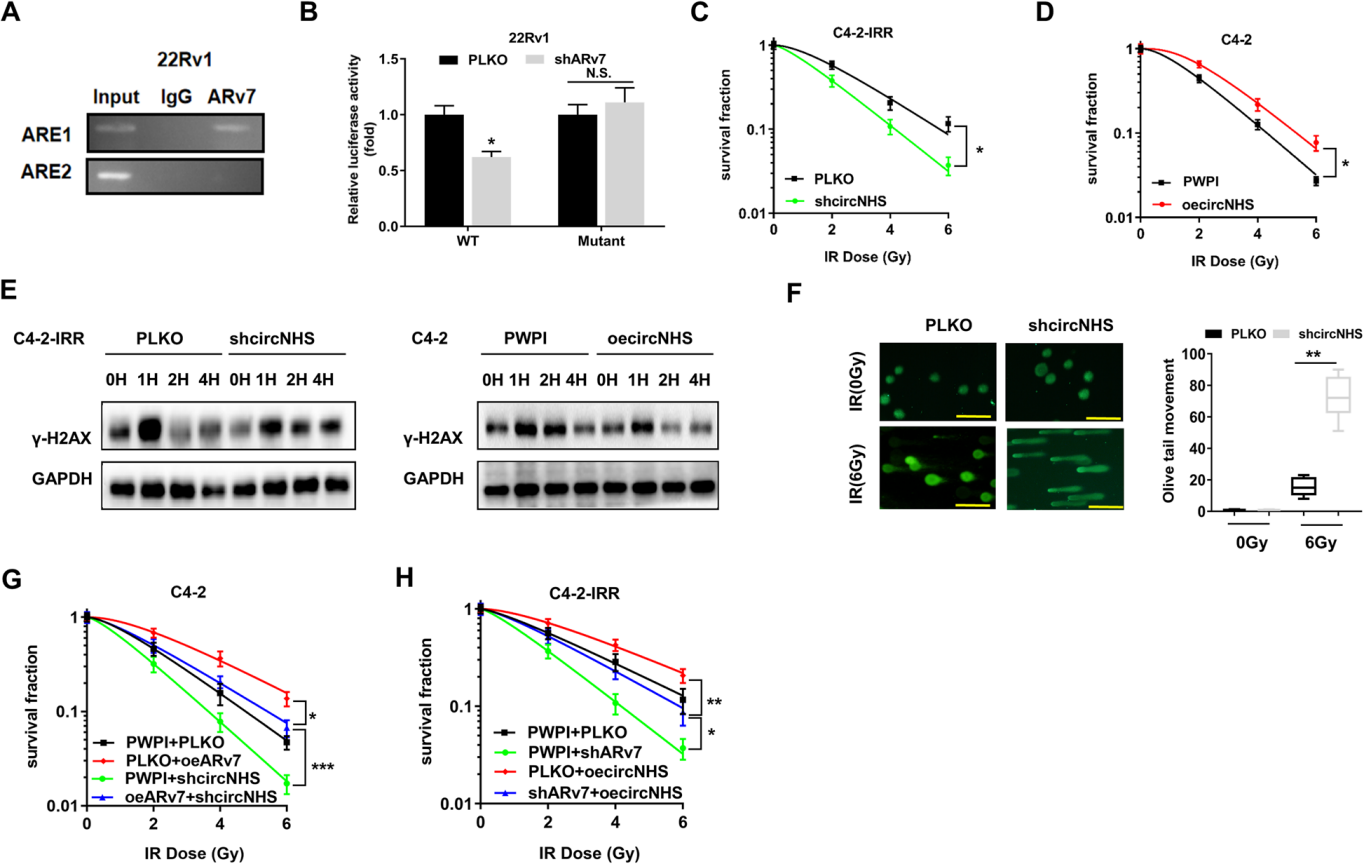
**A** ChIP assay of the two potential ARE binding sites on NHS promoter. **B** Co-transfection of wild-type ARE or mutant NHS promoter pGL3-Luciferase constructs into 22Rv1 cells with/without shARv7. Luciferase activity in 22Rv1 cells carried wild-type or mutant NHS promoter plasmids. **C** shcircNHS effects on C4-2-IRR cells survival after IR using clonogenic assay. **D** The oecircNHS effects on C4-2 cells survival after IR using clonogenic assay. **E** WB analysis of γ-H2AX in different groups. The oecircNHS can decrease DNA damage repair time in C4-2 cells and shcircNHS can increase DNA damage repair time in C4-2-IRR cells. **F** shcircNHS effects on DNA damage in C4-2-IRR cells after IR using comet assay. Scale bar = 10 µm. **G** The oeARv7 and/or shcircNHS effects on C4-2 cell survival after IR using clonogenic assay **H** shARv7 and/or oecircNHS effects on C4-2-IRR cells survival after IR using clonogenic assay. Data are presented as mean ± SEM. *P < 0.05, **P < 0.01, and ***P < 0.001 compared with the controls. N.S., not significant

To further dissect the molecular mechanism of how ARv7 can increase circNHS expression, we examined circNHS transcriptional regulation, and our data indicated that ARv7 could increase NHS expression at both the mRNA and protein levels (Fig. S1D and Fig. S1E). We then searched for potential androgen response elements (AREs) on the NHS 5' promoter region using the JASPAR database. The results revealed that 2 putative AREs were located within 2 kb of the NHS 5' promoter region (Fig. S1F). We then performed chromatin immunoprecipitation (ChIP) in vivo binding assays in 22Rv1 cells, and the results revealed that ARv7 could bind to the ARE1 (Fig. 3A), suggesting that ARv7 might be able to increase circNHS expression via direct binding to a ARE to exert its transcriptional regulation.

We then performed the luciferase reporter assay by inserting the 1 kb 5' promoter region of NHS containing ARE1 into the pGL3 luciferase backbone and also generated a version with a mutated ARE1 (Fig. S1G). The luciferase assay results revealed that decreasing ARv7 by adding shARv7 significantly decreased luciferase activity in 22Rv1 cells transfected with the wild-type NHS promoter construct but not in cells with the mutant NHS promoter construct (Fig. 3B). Importantly, the results from the LinkedOmics database via TCGA data analysis also showed that NHS expression was positively correlated with AR expression in PCa patients (Fig. S1H).

Together, results from Fig. 3A-B suggest that ARv7 increases circNHS expression via altering the transcriptional regulation.

Next, to prove that ARv7 may function by altering circNHS expression to decrease radiosensitivity, we then suppressed the circNHS with the shcircNHS (Fig. S1I), and results revealed that suppressed circNHS led to increase the radiosensitivity in C4-2-IRR cells (Fig. 3C). In contrast, increasing circNHS resulted in decreasing the radiosensitivity in C4-2 cells (Fig. 3D).

The results from the γ-H2AX assay also revealed that decreasing circNHS levels could increase the DNA damage repair time in C4-2-IRR cells (Fig. 3E, left panel). In contrast, increased circNHS expression resulted in less time to complete DNA damage repair in the C4-2 cell line (Fig. 3E, right panel). Similarly, the results from the comet assay also revealed that decreasing circNHS in C4-2-IRR cells significantly increased DNA fragmentation following IR (Fig. 3F).

Finally, the results from the silencing experiment revealed that suppressing circNHS led to a partial reversal of ectopic oeARv7-suppressed radio-sensitivity in the C4-2 cells (Fig. 3G) and increased circNHS led to a partial reversal of the shARv7-increased radio-sensitivity in the C4-2-IRR cells (Fig. 3H).

Together, results from Fig. 3 suggest that ARv7 can function via increasing the circNHS to alter the radiosensitivity.

### Mechanistic dissection of how the circNHS could decrease radiosensitivity: by competing with miR-512-5p/XRCC5 axis

**Fig. 4:**
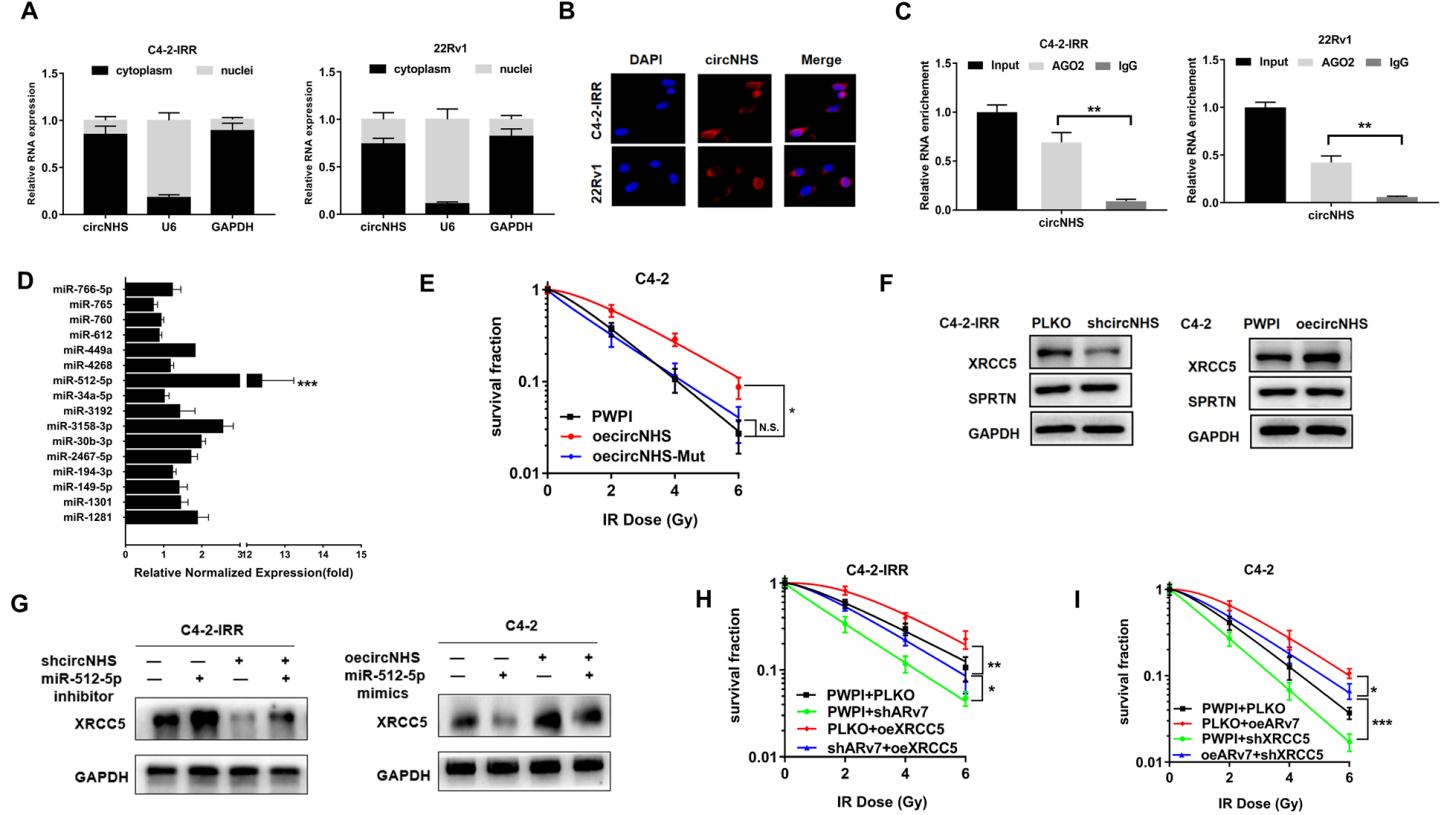
**A** Relative circNHS levels in nuclear and cytosolic fractions of C4-2-IRR cells and 22Rv1 cells are shown. U6 was used as the nuclear control. GAPDH was used as the cytosolic control. **B** RNA-FISH assay results indicated the location of circNHS in C4-2-IRR cells and 22Rv1 cells. **C** RIP experiments were performed using an antibody against AGO2 on extracts from C4-2-IRR cells and 22Rv1 cells. **D** The circRIP was performed in C4-2-IRR cells using a circNHS-specific probe and control probe. The enrichment of miRNAs was detected by RT-qPCR and normalized to the control probe. **E** Mutation of miR-512-5p binding sites abolished the effects of circNHS on radiosensitivity, as revealed using colony formation assay in C4-2 cells. **F** WB analysis of XRCC5 and SPRTN protein levels after shcircNHS in C4-2-IRR cells and oecircNHS in C4-2 cells. **G** WB assays: effects of shcircNHS and/or miR-512-5p inhibitor on XRCC5 protein levels in C4-2-IRR cells (left) and effects of oecircNHS and/or miR-512-5p mimics on XRCC5 protein levels in C4-2 cells (right). **H** shARv7 and/or oeXRCC5 effects on C4-2-IRR cells survival after IR using clonogenic assay. **I** The oeARv7 and/or shXRCC5 effects on C4-2 cell survival after IR using clonogenic assay. *P < 0.05, ^**^P < 0.01, and ^***^P < 0.001 compared with the controls. N.S., not significant. Data are presented as mean ± SEM

Next, to determine how circNHS could decrease radiosensitivity, we first determined the subcellular localization of circNHS in PCa cell lines using a nuclear mass separation assay (Fig. 4A) and FISH analysis (Fig. 4B). The results revealed that circNHS was expressed mainly in the cytoplasm of PCa cells. As early studies indicated that cytoplasmic circRNAs might compete with miRNAs to exert their function [27], we then performed an RNA immunoprecipitation (RIP) assay with an antibody against argonaute 2 (AGO2) in C4-2-IRR and 22Rv1 cells, and the results revealed that circNHS was significantly enriched by the AGO2 antibody (Fig. 4C), suggesting that circNHS might act as a binding platform for AGO2 and miRNAs. Based on these findings, we predicted that circNHS might serve as a binding platform for miRNAs. We then surveyed 3 databases [28, 29] and found that 16 potential miRNAs could bind to circNHS (Fig. 4D). Using probes specifically against circNHS to analyze these 16 candidate miRNAs in the complex, we found only a specific enrichment of miR-512-5p and none of the other rest 15 miRNAs, suggesting that miR-512-5p is one of the circNHS-associated miRNAs in PCa cells.

To further prove that the circNHS can alter radiosensitivity via binding to the miR-512-5p, we then constructed a mutated binding site of miR-512-5p (circNHS-Mut, Fig. S1J). The results from colony formation assays revealed that mutation of the miR-512–5p binding site could completely abolish circNHS-induced radioresistance in C4-2 cells (Fig. 4E).

Recent studies identified 542 ARv7-regulated genes via RNA-seq and CHIP-seq analyses [30]. Among these genes, we found 9 DDR genes and results from targetscan database predicted that XRCC5 and SPRTN can be the potential target genes of miR-512–5p (Fig. S1K). Results from WB analysis demonstrated that circNHS could regulate the expression of XRCC5 protein, and not SPRTN protein levels, suggesting circNHS may function via sponging miR-512–5p to increase the protein expression of XRCC5 (Fig. 4F). As expected, adding miR-512-5p inhibitor led to increase XRCC5 protein expression and could partly reverse the shcircNHS–decreased XRCC5 expression in C4-2-IRR cells (Fig. 4G, left) while adding miR-512–5p mimics led to decreased XRCC5 protein expression and could effectively reverse the oe-circNHS increased XRCC5 expression in C4-2 cells (Fig. 4 G, right).

Results from interruption approaches adding XRCC5-cDNA led to partially reverse the shARv7-increased radiosensitivity in C4-2-IRR cells (Fig. 4H). Similarly, using XRCC5-shRNA further revealed that suppressing XRCC5 led to partially reverse the ARv7-decreased radiosensitivity (Fig. 4I).

Finally, the results from the LinkedOmics database via TCGA data analysis also showed that XRCC5 expression was positively correlated with AR expression in PCa patients (Fig. S1L).

Together, the results from Fig. 4 suggest that circNHS might function by sponging miR-512-5p to alter XRCC5 protein expression and such regulation may then lead to alter the DDR pathway.

### Preclinical studies to target ARv7 with the small molecule of Que increase radiosensitivity

**Fig. 5:**
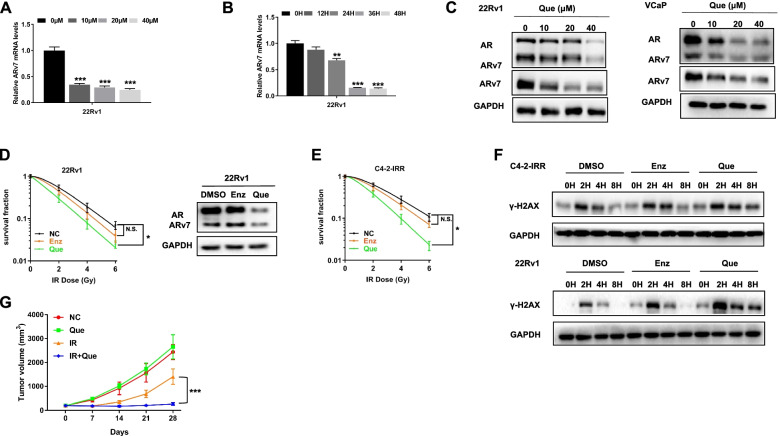
**A** Changes in ARv7 mRNA levels after different Que dose treatments. Control group is the 0 µM group. **B** Changes in ARv7 mRNA after 40 µM Que treatment at different times. Control group is the 0 H group. **C** Changes in AR and ARv7 protein levels in 22Rv1 and VCaP cells after different Que dose treatment. **D** Que and Enz effects on 22Rv1 cells survival after IR using clonogenic assay. **E** Que and Enz effects on C4-2-IRR cells survival after IR using clonogenic assay. **F** WB analysis of γ-H2AX levels in DMSO, Enz and Que groups after 4 Gy IR. **G** The average tumor volumes of the in vivo mouse model (each group *N* = 6) studies were recorded every 7 days and tumor volume (mm^3^) changes were graphed. Data are presented as mean ± SEM. *P < 0.05, **P < 0.01, ***P < 0.001 compared with the controls. N.S., not significant

All above results suggest that IR may have unwanted side effects of inducing the ARv7 expression to increase the radioresistance. We are interested to see if adding small molecules can have similar effect to suppress the ARv7 expression. We are interested to test the Que, as our previous studies indicated that Que could suppress AR mRNA and protein expression [31]. Here we found that adding Que could also significantly decrease the ARv7 expression at both mRNA and protein levels in 22Rv1 and VCaP cells (Fig. 5A-C).

Furthermore, adding 40 µM Que to reduce both AR and ARv7 expression can then lead to increase the radiosensitivity to better suppress the C4-2-IRR and 22Rv1 cell growth using clonogenic assay (Fig. 5D-E). Mechanism dissection revealed that adding Que, and not Enz, could decrease DNA damage repair via detecting γ-H2AX levels (Fig. 5F).

To further validate these in vitro findings in the in vivo mouse model, we established subcutaneous 22Rv1 xenografts in nude mice. Results showed that combined IR treatment with Que treatment (75 mg/kg) led to suppress the tumor growth approximately 70% to 75% on day 28, as compared to IR alone or Que alone (Fig. 5G).

Together, results from Fig. 5 suggest that the small molecule Que can increase radiosensitivity to better suppress the PCa cells growth via targeting both ARfl and ARv7 levels.

## Discussion

Studies reported that AR-full-length mRNA is coded from eight exons and the protein contains four functional domains: an N-terminal domain (NTD), a central DNA-binding domain (DBD), a short hinge region and a C-terminal ligand-binding domain (LBD) [32]. Constitutively active ARVs have now been discovered [33], most of which contain the NTD and the DBD, but lack the LBD due to a truncation by a cryptic exon. As conventional ADT inhibits androgen-dependent activation of AR, the presence of C-terminal truncated ARVs provides a compelling mechanism for CRPC cells to circumvent ADT [34]. ARVs are nuclear, constitutively active, and bind to similar AREs as full-length AR [34]. More than 20 different ARVs that have been identified in clinical PCa tissues and PCa cell lines and among these variants, AR-v7, originates from contiguous splicing of AR exon 1, exon 2, and exon 3 with the cryptic exon 3 (CE3) present within the canonical intron 3 of the AR gene. It is one of the most well characterized ARVs and can be reliably measured in tissue and liquid biopsy specimens, and blood-based detection [35–38]. Due to lack of the LBD, ARv7 can circumvent the pharmacological effects of second-generation antiandrogens such as Enz, which targets LBD of AR directly. Besides, ARv7 could be induced by ADT treatment and also serves as a predictive biomarker for response to Enz or abiraterone acetate [7, 9, 23].

We and some groups have demonstrated that AR expression and activity are upregulated following IR in PCa models and AR can activate DDR pathways [4, 5, 10, 39, 40] in PCa, providing rationale for concurrent ADT + RT therapy [6, 41] as being better than RT therapy alone. These preclinical studies also have led to testing combinations of RT with next generation hormonal agents such as abiraterone, Enz, apalutamide and AR degradation enhancer, ASC-J9 [40, 42–45]. However, despite use of combined ADT and RT, the recurrence rate is up to 50% in high-risk PCa patients [6]. To dissect this mechanism, we focus on the role of ARv7 expression and activity in PCa after RT and radioresistant PCa cells. Our in vitro and in vivo experiments results suggest that ARv7 is highly expressed in radioresistant PCa cells and IR could induce ARv7 expression in PCa which increases the clonogenic survival of PCa cells after irradiation. Moreover, the IR-induced ARv7 could mediate PCa DDR and is not affected by Enz treatment. Thus, our study may explain the clinical phenomenon that many patients first respond to RT + ADT then eventually develop biochemical failure and that patients with ARv7 expression might not be eligible for such a combination treatment. Similarly, Yin et al. also found that ARVs alone can regulate DDR following PCa RT. To clarify these issues, further investigations on PCa RT clinical samples are necessary.

To further dissect the mechanism how ARv7 may alter the IR-sensitivity, recently studies found that circRNAs may play important roles in radiosensitivity. Several studies have revealed that circRNA levels might be substantially altered after IR, including esophageal cancer [3], cervical cancer [46], glioma [47], nasopharyngeal carcinoma [48], oral squamous cell carcinoma [49], colorectal cancer [50] and hepatocellular carcinoma [51]. Previously, we found that circZEB1 was upregulated after IR in PCa cells and might function as a sponge for miR-141-3p [22]. In this study, we analyzed our radioresistant C4-2 cell models and analyzed the twenty most altered circRNAs from circRNA-Array data [25]. Studies showed that ARv7 is constitutively active and capable of activating genes even in the absence of androgen [52]. Our results showed that only circNHS could be regulated by ARv7 among these twenty circRNAs. By gain/loss of function assays of circNHS, we found that it could partially reverse the effects of ARv7 on PCa radiosensitivity. Moreover, there are currently no other published reports about circNHS functions. To our knowledge, this is the first study about circRNA function in radioresistant PCa cells to date, which might contribute to a better understanding of ARv7 function in RT.

Taken together, a requirement for inhibiting both full-length AR and ARv7 transcriptional activities is necessary to effectively improve radiosensitivity. To target both the IR-induced AR and ARv7 with a small molecules, which we screened and found that a small molecule of Que which is a flavonoid widely found in vegetables and fruits particularly in onions, apples, and red wines [12] could be effective. Moreover, Que has very low toxicity and rarely produces any side effects even at high dose of 200 mg/kg given to rats and mice [53, 54], and a clinical trial showed that a total consumption of 1000 mg Que per day could be well tolerated in human and is not associated with any side effects [55, 56]. Recent studies have found its antitumor role in many cancers and that it can function as a radiosensitizer in many cancers [57–59]. Our previous study also revealed that Que inhibited LNCaP cells growth through inhibiting AR expression and its inducible genes [31]. In this study, we found that Que could inhibit the transcriptional activities of both full-length AR and ARv7 and enhance RT-mediated cell killing compared with second-generation antiandrogens such as Enz. Thus, we provide proof-of-principle pre-clinical in vitro and in vivo evidence to rationalize the clinical use of Que to enhance the effect of IR as a potential strategy to improve the outcomes of PCa patients who undergo RT. Indeed, further randomized clinical trials are required to assess the effect of combining Que and RT for PCa patients.

## Conclusions

In summary, our research here demonstrated that IR could induce ARv7 in PCa cells and IR-induced ARv7 could decrease the radiosensitivity through mediating circNHS/miR-512-5p/XRCC5 pathway. Targeting the IR-induced ARv7 and AR with the small molecule of Que, could increase radiosensitivity to better suppress the PCa cells. These findings can provide a solid foundation for the clinical use of Que in RT and raise the possibility of applying combined use of RT and Que to gain better clinical results than RT + ADT treatment.

## Supplementary Information

**Additional file 1:** **Supplementary Fig. 1A.** C4-2 parental cells and C4-2-IRR cells were cultured with DMSO or 5 µM Enz for 24 h, then treated with escalating doses of IR. **B** WB analysis of AR and ARv7 levels in C4-2 parental cells and C4-2-IRR cells. **C** Relative circNHS levels in high-grade (Gleason > 8) and low-grade (Gleason < 6) PCa tissues. **D** shARv7 on C4-2-IRR cells to detect NHS mRNA and protein levels. **E** oeARv7 on C4-2 cells to detect NHS mRNA and protein levels. **F** Two potential AREs on NHS 2 kb promoter region. **G** Diagram of cloning the 2 kb NHS promoter into pGL3 basic luciferase report vector. Site-directed mutagenesis of ARE1 was done by mutating part of the ARE sequence into Xba1 (–TCTAGA–) cutting site. **H** NHS expression was positively correlated with AR expression as shown by TCGA analysis. **I** Relative RNA levels after transfection with shcircNHS or oecircNHS. **J **Site-directed mutagenesis of circNHS was done by mutating binding sites sequences. **K** Overlapping of the potential target genes of circNHS predicted by three databases. **L** XRCC5 expression was positively correlated with AR expression by TCGA database. Data are presented as mean ± SEM. *P < 0.05, *P < 0.01, *P < 0.001 compared with the controls. N.S., not significant

**Additional file 2.**

## Data Availability

The datasets used and/or analyzed during this study are available from the corresponding author on reasonable request.

## Abbreviations

ceRNA: Competing endogenous RNA
CircRNA: Circular RNA
PCa: Prostate cancer
qRT-PCR: Real-time quantitative polymerase chain reaction
miRNA: MicroRNA
RIP: RNA immunoprecipitation
RNA-seq: RNA sequencing
TCGA: The Cancer Genome Atlas
Que: Quercetin
AR: Androgen receptor
ARv7: Androgen receptor variant 7
