# Supplementary figures and images for: Targeting the radiation-induced ARv7-mediated circNHS/miR-512-5p/XRCC5 signaling with Quercetin increases prostate cancer radiosensitivity
Source: J Exp Clin Cancer Res. 2022 Aug 3;41:235. doi: 10.1186/s13046-022-02287-4 (PMC9347162; doi:10.1186/s13046-022-02287-4)

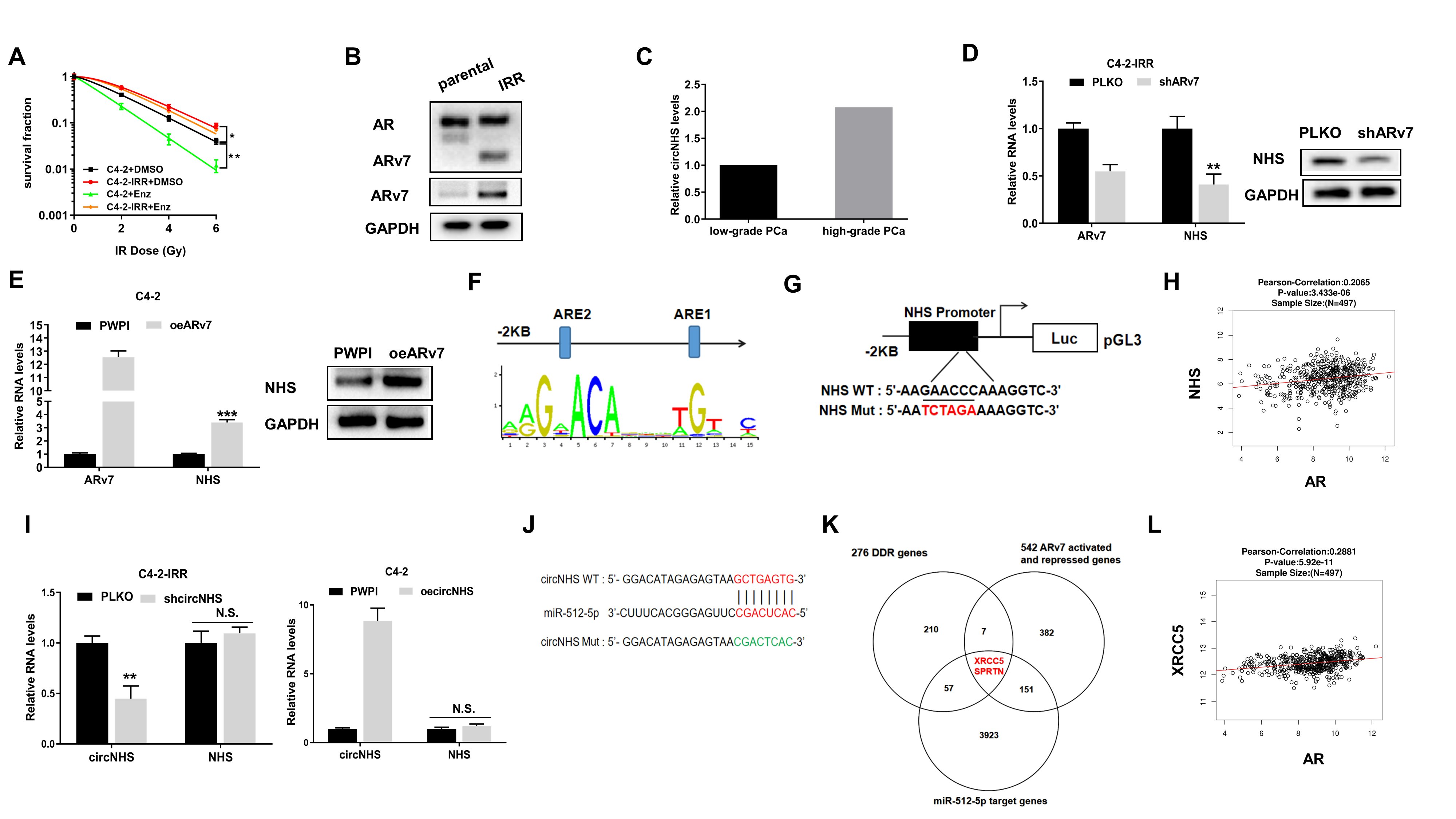

Supplement: Supplementary file 1 — Additional file 1: Supplementary Fig. 1A. C4-2 parental cells and C4-2-IRR cells were cultured with DMSO or 5 µM Enz for 24 h, then treated with escalating doses of IR. B WB analysis of AR and ARv7 levels in C4-2 parental cells and C4-2-IRR cells. C Relative circNHS levels in high-grade (Gleason > 8) and low-grade (Gleason < 6) PCa tissues. D shARv7 on C4-2-IRR cells to detect NHS mRNA and protein levels. E oeARv7 on C4-2 cells to detect NHS mRNA and protein levels. F Two potential AREs on NHS 2 kb promoter region. G Diagram of cloning the 2 kb NHS promoter into pGL3 basic luciferase report vector. Site-directed mutagenesis of ARE1 was done by mutating part of the ARE sequence into Xba1 (–TCTAGA–) cutting site. H NHS expression was positively correlated with AR expression as shown by TCGA analysis. I Relative RNA levels after transfection with shcircNHS or oecircNHS. J Site-directed mutagenesis of circNHS was done by mutating binding sites sequences. K Overlapping of the potential target genes of circNHS predicted by three databases. L XRCC5 expression was positively correlated with AR expression by TCGA database. Data are presented as mean ± SEM. *P < 0.05, *P < 0.01, *P < 0.001 compared with the controls. N.S., not significant [file 13046_2022_2287_MOESM1_ESM.tif]
