# Supplementary material for: Targeting the radiation-induced ARv7-mediated circNHS/miR-512-5p/XRCC5 signaling with Quercetin increases prostate cancer radiosensitivity
Source: J Exp Clin Cancer Res. 2022 Aug 3;41:235. doi: 10.1186/s13046-022-02287-4 (PMC9347162; doi:10.1186/s13046-022-02287-4)
Supplement: Supplementary file 2 — Additional file 2. [file 13046_2022_2287_MOESM2_ESM.doc]

**Table 1 Quantitative RT-PCR primers**

| ARv7 | Forward | CCATCTTGTCGTCTTCGGAAATGTTA |
| --- | --- | --- |
|  | Reverse | TTTGAATGAGGCAAGTCAGCCTTTCT |
| NHS | Forward | CAGCAGCGCAACATCTTCC |
|  | Reverse | CGGTCCTTAAACTCACGCCT |
| GAPDH | Forward | GGAGCGAGATCCCTCCAAAAT |
|  | Reverse | GGCTGTTGTCATACTTCTCATGG |
| hsa_circ_0000119 | Forward | TTGGCTGAGAAACTCCTTCC |
|  | Reverse | GCTTCTTCCAAGGCCTTCTC |
| hsa_circ_0000518 | Forward | GCCCTAACAGGGCTCTCC |
|  | Reverse | AAGGGACATGGGAGTGGAGT |
| hsa_circ_0000554 | Forward | GGCACTAGGGAGGGACTCAT |
|  | Reverse | GGGCAGAGACAGAGTGGATG |
| hsa_circ_0001313 | Forward | CGAGACAGACGACGACAAAA |
|  | Reverse | TTGACGGTCATCTTCTATTTGC |
| hsa_circ_0002019 | Forward | GGCTTCAGAAAAGGATGCTG |
|  | Reverse | TGAAAAGAGGGAGAGCTGGA |
| hsa_circ_0004370 | Forward | CAGGAGACGTGACTGCTGTG |
|  | Reverse | TGTCCTATTCCTTCGCTGCT |
| hsa_circ_0004519 | Forward | TGGCATACTGTGATGCTCTG |
|  | Reverse | CGGCACTGTGCTGATTCTAA |
| hsa_circ_0007380 | Forward | GTTAGCATGATCCCACCACTG |
|  | Reverse | TTTTCCAAGTGTGGCGATTT |
| hsa_circ_0007613 | Forward | GTCCCGTGTGGTCCGAAA |
|  | Reverse | CTGCCGTCCTTCACAACTTC |
| hsa_circ_0008301 | Forward | CTTCCAGCTGCCATGGTG |
|  | Reverse | CGTGATGCGGAGGAAGTC |
| hsa_circ_0013737 | Forward | GGAGGCCTACTTGCAGCATA |
|  |  |  |
|  | Reverse | CCATCTTTGCCCATCTAGGA |
| hsa_circ_0014879 | Forward | GGCACTGGAACCAGACTCTC |
|  | Reverse | GTAGCCAGCCTTTCCTCCTT |
| hsa_circ_0029853 | Forward | GACCTTGCAAAGGAGGTTCA |
|  | Reverse | ATCTTGGGGGAATGAAGAGG |
| hsa_circ_0032832 | Forward | TCATCAATGCTGAGGAGCAG |
|  | Reverse | CTTCAGAATGGGTGGTCCAG |
| hsa_circ_0035381 | Forward | CTGTGCTGTGGACACTGCTT |
|  | Reverse | TACCATGTGAACCAGGAGCA |
| hsa_circ_0058058 | Forward | GCAGAGCTCCGAGAGTAAGG |
|  | Reverse | GAAGCGACCAGATTCAAACC |
| hsa_circ_0061137 | Forward | GCCAGAAAAGACTGCTGCTC |
|  | Reverse | GTTGTCAGCCACTGTTCACG |
